# Cost-utility analysis of robotic and conventional total knee arthroplasty: A 200-patient micro-costing study in a public sector institution

**DOI:** 10.1007/s11701-026-03658-1

**Published:** 2026-07-20

**Authors:** Warran Wignadasan, Andreas Fontalis, Abdulellah Alsheddi, Jenni Tahmassebi, Nishma Patel, Elena Pizzo, Fares S. Haddad

**Affiliations:** 1 Department of Trauma and Orthopaedic Surgery, University College Hospital, 235 Euston Road, Fitzrovia, London, NW1 2BU UK; 2 Division of Surgery & Interventional Science, University College London, Gower Street, London, WC1E 6BT UK; 3 Department of Primary Care and Population Health, University College London, Gower Street, London, WC1E 6BT UK; 4 Department of Orthopaedic Surgery, The Princess Grace Hospital, 42- 52 Nottingham Pl, Marylebone, London, W1U 5NY UK

## Abstract

Robotic-arm assisted total knee arthroplasty (RO TKA) has been developed to improve surgical precision and reproducibility compared with conventional techniques. However, the economic implications of adopting robotic technology remain uncertain, particularly within publicly funded healthcare systems. This study aimed to evaluate the in-hospital costs and health-related quality of life outcomes associated with RO TKA compared with conventional TKA (CO TKA) using a cost-utility framework within a UK National Health Service (NHS) institution. A propensity-matched cohort cost-utility analysis was performed including 200 patients undergoing primary TKA for end-stage osteoarthritis at a single high-volume tertiary centre. 100 patients underwent RO TKA and 100 underwent CO TKA, matched 1:1 for age, sex and American Society of Anesthesiologists (ASA) grade. Direct in-hospital costs were obtained using a micro-costing approach. Health-related quality of life was measured using the EQ-5D-3 L preoperatively and at one year postoperatively to estimate quality-adjusted life years (QALYs). The primary outcome was the incremental cost-effectiveness ratio (ICER). Deterministic and probabilistic sensitivity analyses were performed to assess uncertainty. Mean total in-hospital costs were higher for RO TKA compared with CO TKA (£6,517 vs. £6,204), representing an incremental cost of £313 per patient. Patients undergoing RO TKA demonstrated a significantly shorter length of stay (2.3 vs. 3.1 days; *p* = 0.008) and lower ward (*p* = 0.029) and drug and pharmacy (*p* = 0.041) costs. One-year postoperative EQ-5D-3 L scores were higher in the RO TKA cohort (0.82 vs. 0.75; *p* < 0.001), resulting in a greater mean QALY gain (0.192 vs. 0.140). The incremental QALY gain of 0.052 produced an ICER of £6,019 per QALY gained, well below the National Institute for Health and Care Excellence (NICE) willingness-to-pay threshold of £20,000–£30,000 per QALY. Sensitivity analyses confirmed the robustness of these findings. RO TKA demonstrated improved postoperative health utility and is likely to achieve cost-effectiveness despite modestly higher upfront costs. Within a publicly funded healthcare system, RO TKA is likely to represent a clinically and economically viable innovation in knee arthroplasty, under the costing assumptions applied in this analysis.

## Key Points

Robotic-assisted total knee arthroplasty was associated with slightly higher upfront hospital costs than conventional surgery but resulted in better patient quality-of-life outcomes one year after surgery. When both costs and health benefits were considered together, robotic surgery was cost-effective within commonly accepted NHS thresholds. These findings suggest robotic technology may represent a valuable innovation for publicly funded healthcare systems.

## Introduction

The rising prevalence of knee osteoarthritis, driven by population aging, increased longevity and higher functional expectations, continues to accelerate demand for total knee arthroplasty (TKA) [1, 2]. TKA is widely regarded as a reliable and cost-effective procedure for end-stage arthritis of the knee, providing substantial pain relief and improvements in quality of life [3]. Despite its success, up to 10–15% of patients remain dissatisfied following surgery, often as a result of persistent pain, stiffness or functional limitations [4, 5]. Malalignment, ligament imbalance and variability in implant positioning have been recognised as potential contributors to suboptimal outcomes and early failures [6–8].

These challenges have spurred interest in technological innovations aimed at improving surgical precision and reproducibility. Robotic-arm assisted TKA (RO TKA) has emerged as one such development, offering enhanced accuracy of implant positioning, intraoperative optical motion capture technology and the capacity to achieve more reliable alignment and soft tissue balancing compared to conventional TKA (CO TKA) [9–12]. The extent to which these technical advances translate into improved long-term clinical outcomes and cost-effectiveness has yet to be conclusively established.

As healthcare systems move increasingly toward value-based models, the economic implications of adopting novel technologies, such as robotic assistance in arthroplasty, have been central to policy and investment decisions [13]. Robotic systems require substantial financial outlay, including acquisition costs, maintenance, disposables and additional preoperative imaging. These expenses must be balanced against the potential benefits reported such as reduced length of stay (LOS) [14, 15], reduced soft tissue trauma [16] and improved early functional recovery [10]. Economic evaluations comparing RO TKA and CO TKA remain sparse and are predominantly derived from private or mixed healthcare systems, where care pathways, patient populations and resource allocation differ substantially from those in publicly funded settings.

There is a particular need for robust micro-costing based analyses conducted within publicly funded healthcare institutions, where there is a paucity of evidence on the cost-effectiveness of robotic-assisted arthroplasty. To address this gap, this study aims to evaluate the in-hospital costs and health-related quality of life outcomes of RO TKA versus CO TKA across a one-year time horizon within a high-volume UK National Health Service (NHS) institution. This study reports a comprehensive cost-utility analysis of 200 TKAs (100 robotic and 100 matched conventional cases) and to our knowledge, represents the largest evaluation of its kind performed exclusively within a publicly funded healthcare setting.

## Methods and materials

### Study overview

We conducted a propensity-matched cohort cost-utility analysis to compare RO TKA with CO TKA within a single high-volume tertiary arthroplasty centre operating in a publicly funded healthcare system. The evaluation adhered to established economic evaluation standards, using quality-adjusted life years (QALYs) as a measure of health benefit in accordance with National Institute for Health and Care Excellence (NICE) guidance [17]. Analyses were performed from the perspective of the UK NHS using 2023/2024 UK pounds sterling over a time horizon of one year. Institutional approval was obtained prior to data collection and analysis.

### Study population

A total of 200 patients (100 in the robotic arm and 100 in the conventional arm) undergoing primary TKA for end-stage osteoarthritis were included in the study. Patients were identified from all primary elective TKAs performed for end-stage osteoarthritis at our tertiary academic centre during the study period. Robotic-assisted and conventional procedures were undertaken during the same operative time window, and cases were subsequently propensity matched 1:1 for age, sex and American Society of Anesthesiologists (ASA) grade to minimise baseline differences and reduce potential selection bias. All surgical procedures were performed by fellowship-trained, arthroplasty surgeons utilizing a standard midline approach. All RO TKA procedures were performed using the MAKO robotic arm system (Stryker, Kalamazoo, MI, USA) with preoperative planning and intraoperative execution based on a functional alignment strategy. Conventional procedures were undertaken using standard mechanical instrumentation. A cemented posterior-stabilized (PS) Triathlon (Stryker, Kalamazoo, MI, USA) TKA prosthesis was implanted in all of the study participants.

### Resource use and costing

A comprehensive micro-costing methodology was used to capture all direct hospital costs that were incurred during the primary admission from the hospital’s costings department. Costed items included theatre costs (staffing and overheads), anaesthetic services, postoperative recovery care, inpatient ward costs (including physiotherapy costs), blood product costs, laboratory investigation costs, robotic disposables for RO TKA cases and drug and pharmacy costs. Costs for postoperative imaging were also incorporated.

The MAKO robotic platform costs were sourced directly from the manufacturer. Two procurement options were available: a one-off purchase or a subscription model priced at £11,000 monthly per robotic system, which covers maintenance and onsite intraoperative support from a MAKO Product Specialist (MPS). For our analysis, the monthly subscription model was applied. During the 3-month period in which 100 consecutive RO TKAs were performed, two robotic systems were in continuous use within the department, resulting in a total leasing cost of £66,000. Over the same interval, an additional 85 robotic procedures were completed (45 total hip arthroplasties (THA) and 40 partial knee arthroplasties (PKA)). When this total activity was used to distribute the expenditure, the capital allocation was £357 per robotic case, which was applied to each RO TKA. RO TKA required a preoperative CT scan in accordance with manufacturer protocol (NHS tariff £69 [18]) in addition to robotic-specific disposable instrumentation (£286 per case). Capital depreciation costs were not included owing to the short analytic time horizon.

### Health utility measurement

Health-related quality of life (QoL) was measured using the EuroQol 5 Dimensions, 3 Levels (EQ-5D-3 L) instrument administered preoperatively and again at one year following surgery. Utility values were generated using the EQ-5D-3 L value set in line with National Institute for Health and Care Excellence (NICE) recommendations [19]. QALYs were then estimated for each patient using an area-under-the-curve (AUC) approach, applying linear approximation where changes in health status are gradual over time.

### Economic analysis

The primary endpoint was the incremental cost-effectiveness ratio (ICER), defined as the difference in mean total cost between RO TKA and CO TKA divided by the corresponding difference in mean QALYs. Uncertainty surrounding the mean estimate was assessed through non-parametric bootstrapping with 10,000 iterations, with results displayed on a cost-effectiveness plane. Deterministic sensitivity analyses (DSA) varied major cost components - inpatient ward costs, operating theatre costs, robotic capital and drug and pharmacy costs - by ± 20%. A probabilistic sensitivity analysis (PSA) was also undertaken, applying gamma distributions to cost inputs and beta distributions to utility values. Key model input parameters used in the cost-utility analysis, including cost inputs, utility values and willingness-to-pay (WTP) thresholds are summarised in Table 1.

**Table 1:** Key input parameters and base-case values used in the cost-utility analysis

Parameter	Base value	Distribution used in sensitivity analysis	Source
**Mean cost per patient - RO TKA (£)**	6,517	Gamma	Micro-costing analysis of study cohort
**Mean cost per patient - CO TKA (£)**	6,204	Gamma	Micro-costing analysis of study cohort
**Incremental cost (£)**	313	Derived	Difference between groups
**Preoperative EQ-5D-3 L utility - RO TKA**	0.44	Beta	Study cohort
**Preoperative EQ-5D-3 L utility - CO TKA**	0.45	Beta	Study cohort
**Postoperative EQ-5D-3 L utility - RO TKA**	0.82	Beta	Study cohort
**Postoperative EQ-5D-3 L utility - CO TKA**	0.75	Beta	Study cohort
**Mean QALY gain - RO TKA**	0.192	Beta	Area-under-the-curve method
**Mean QALY gain - CO TKA**	0.140	Beta	Area-under-the-curve method
**Incremental QALY gain**	0.052	Derived	Difference between groups
**Robotic capital cost per case (£)**	357	Gamma	MAKO leasing model allocation
**Robotic disposable cost per case (£)**	286	Gamma	Manufacturer pricing
**Preoperative CT cost (£)**	69	Gamma	NHS tariff
**Mean LOS - RO TKA (days)**	2.5	Gamma	Study cohort
**Mean LOS - CO TKA (days)**	3.3	Gamma	Study cohort
**Ward cost per admission (£)**	2,161 (RO) / 2,801 (CO)	Gamma	Micro-costing data
**Theatre cost per case (£)**	1,942 (RO) / 1,657 (CO)	Gamma	Micro-costing data
**Drug and pharmacy cost (£)**	439 (RO) / 529 (CO)	Gamma	Micro-costing data
**Willingness-to-pay threshold (£ per QALY)**	£20,000–£30,000	Fixed	NICE guidance

LOS = length of stay; CT = computer tomography; EQ-5D-3 L = EuroQol 5 Dimensions, 3 Levels; RO TKA = Robotic-arm assisted total knee arthroplasty; CO TKA = conventional total knee arthroplasty; QALY = quality-adjusted life years

*Mann-Whitney U test

### Statistical analysis

Normality of continuous variables were evaluated with the Shapiro-Wilk test, followed by independent t-tests or Mann-Whitney U tests for group comparisons. Categorical variables were compared using Chi-squared or Fisher’s exact tests. Statistical significance was defined as a two-tailed *p* < 0.05. Cost data were summarized using mean values for economic comparisons, consistent with standard health economic methodology, with medians and interquartile ranges (IQRs) reported to reflect skewed distributions [20]. All analyses were performed using SPSS version 30.0 (IBM Corp., Armonk, NY, USA) and R version 4.3.0 (R Foundation for Statistical Computing, Vienna, Austria).

## Results

The study cohort comprised 200 patients, including 100 who underwent CO TKA and 100 who underwent RO TKA. No statistically significant differences were observed between groups in terms of age (*p* = 0.481), sex (*p* = 0.884), BMI (*p* = 0.415), ASA score (*p* = 0.541) or anaesthetic agent used (*p* = 0.322) (Table 2). None of the study participants needed further operative intervention for any postoperative complications.

**Table 2:** Demographic data

Demographics	RO TKA	CO TKA	*P*-value
Mean age, yrs (range)	65 (44 to 80)	63 (43 to 80)	0.481*
Sex, n (%)			0.884*†*
Female	57 (57.0)	55 (55.0)	
Male	43 (43.0)	45 (45.0)	
Mean BMI, kg/m^2^ (SD)	27.5 (4.0)	26.8 (4.2)	0.415*
ASA grade, n (%)			0.541*†*
I	22 (22.0)	18 (18.0)	
II	64 (64.0)	67 (79.0)	
III	14 (14.0)	15 (15.0)	
Anesthesia, n (%)			0.322*†*
General	59 (59.0)	51 (51.0)	
Spinal	41 (41.0)	49 (49.0)	

ASA = American Association of Anesthesiologists; BMI = body mass index; RO TKA = Robotic-arm assisted total knee arthroplasty; CO TKA = conventional total knee arthroplasty

*Mann-Whitney U test  †Chi-squared test

### Base case analysis - cost outcomes

During the study period, aggregate in-hospital costs were higher for the RO TKA cohort compared with the CO TKA cohort (*£*651,674 vs. *£*620,416), equating to a greater mean per-patient cost *(*£6,517 vs. £6,204). When robotic-specific costs, including fixed capital expenditure, consumables and preoperative CT scanning, were excluded, RO TKA demonstrated a lower mean total cost per patient than CO TKA (£5,805 vs. £6,204) (Table 3).

**Table 3:** Comparison of outcome metrics between RO TKA and CO TKA

Outcome Metric	RO TKA Median [IQR]	RO TKA Mean (SD)	CO TKA Median [IQR]	CO TKA Mean (SD)	Difference in mean cost (£) (RO – CO) (bootstrapped 95% CI)	*P*-value
**LOS (days)**	2.3 [1.8–3.1]2	2.5 (1.1)	3.1 [2.3–4.1]	3.3 (1.6)	-	0.008*
**Pre-operative CT scan cost (£)**	69 [69–69]	69	0 [0–0]	0 (0)	69 (69 to 69)	< 0.001*
**Ward costs (£)**	2,123 [1,617 − 2,686]	2,161 (925)	2,480 [1,949–3,398]	2,801 (1,245)	-589 (-1,040 to -160)	0.029*
**Theatre costs (£)**	1,971 [1,681 -2,178]	1,942 (352)	1,663 [1,378–1,865]	1,657 (358)	285 (169 to 402)	< 0.001*
**Anaesthetic costs (£)**	587 [489–718]	644 (238)	592 [495–735]	639 (212)	4 (-69 to 79)	0.378*
**Recovery costs (£)**	415 [317–6592]	452 (190)	462 [334–559]	474 (187)	-22 (-84 to 41)	0.315*
**Drug & pharmacy costs (£)**	447 [411–464]	439 (72)	503 [444–550]	529 (91)	-79 (-133 to -29)	0.041*
**Pathology costs (£)**	12 [12–24]	25 (22)	12 [12–24]	31 (58)	-5 (-20 to 7)	0.428*
**Blood transfusion costs (£)**	0 [0–0]	1 (3)	0 [0–0]	1 (4)	0 (-1 to 1)	0.662*
**Radiology costs (£)**	65 [65–65]	70 (26)	65 [65–65]	72 (34)	-4 (-16 to 7)	0.747*
**Robotic consumables (£)**	286 [286–286]	286 (0)	0 [0–0]	0 (0)	286 (286 to 286)	< 0.001*
**Total cost** **including robotic capital costs (£)**	6,371 [5,670–7,193]	6,517 (1,267)	5,958 [4,900–7,155]	6,204 (1,712)	62 (-621 to 496)	0.013*
**Pre-op EQ-5D-3 L**	0.46 [0.41–0.53]	0.44 (0.15)	0.47 [0.42–0.54]	0.45 (0.13)	-	0.541*
**Post-op EQ-5D-3 L**	0.83 [0.71–0.91]	0.82 (0.13)	0.77 [0.63–0.88]	0.75 (0.17)	-	< 0.001*

LOS = length of stay; IQR = interquartile range; CT = computer tomography; EQ-5D-3 L = EuroQol 5 Dimensions, 3 Levels; RO TKA = Robotic-arm assisted total knee arthroplasty; CO TKA = conventional total knee arthroplasty

*Mann-Whitney U test

The median LOS was significantly shorter for the RO TKA cohort at 2.3 days (interquartile range (IQR) 1.8 to 3.1) compared with 3.1 days (IQR 2.3 to 4.1) in the CO TKA group (*p* = 0.008), representing a mean difference of 0.8 days (Cohen’s d = 0.58, medium effect size). This finding was reflected in the ward-related costs, with the RO TKA cohort demonstrating significantly lower mean ward costs than the CO TKA cohort (*p* = 0.029) (Table 3). Consistent with these findings, drug and pharmacy costs were significantly lower in the RO TKA group than in the CO TKA group (£449 vs. £529; *p* = 0.041).

Theatre costs differed significantly between the two cohorts, with the RO TKA group incurring a higher mean cost per case (£1,942 vs. £1,657; *p* < 0.001). The difference observed was largely driven by increased overall operating times associated with the RO TKA procedures. Moreover, robotic consumables, accounting for £286 per case, added costs to each of the robotic cases. We found no statistically significant differences in mean postoperative pathology costs between the cohorts (£25 in the robotic group and £31 in the conventional group; *p* = 0.428). There were also no significant differences in cost in mean postoperative recovery (*p* = 0.315), blood transfusion (*p* = 0.662) and radiology (*p* = 0.747) costs.

### Health utility and QALY outcomes

There were no significant differences between the preoperative EQ-5D-3 L utility scores between the two groups (RO TKA mean 0.44 vs. CO TKA 0.45; *p* = 0.541). At one year postoperatively, the RO TKA cohort demonstrated higher mean EQ-5D-3 L score compared to the conventional cohort (0.82 vs. 0.75; *p* < 0.001), representing a between-group difference of 0.07 (Cohen’s d = 0.46). Mean QALY gain over one year, calculated using a baseline-adjusted area-under-the-curve (AUC) approach at the individual level was 0.140 (95% CI 0.121–0.158) in the control group and 0.192 (95% CI 0.175–0.209) in the robotic group. The mean difference in QALY gain between the two groups was 0.052 (95% CI 0.027–0.078; *p* < 0.001).

### Incremental cost-effectiveness analysis

RO TKA was associated with a higher mean total cost per patient compared with CO TKA (£6,517 vs. £6,204), corresponding to an incremental cost of £313 per patient. In conjunction with an incremental QALY gain of 0.052, the resulting ICER amounted to £6,019 per QALY gained. This value lies well below the accepted NICE WTP threshold of £20,000 - £30,000 per QALY.

### Deterministic sensitivity analysis

DSA demonstrated that the base-case ICER for RO TKA was robust to plausible variation in key cost inputs. One-way sensitivity analyses were performed by varying ward costs, theatre costs, robotic capital costs and drug and pharmacy costs by ± 20% from their base-case values. Across all scenarios tested, the ICER remained well below the NICE WTP threshold of £20,000 per QALY, with values ranging approximately between £4,500 and £8,000 per QALY.

**Fig. 1:**
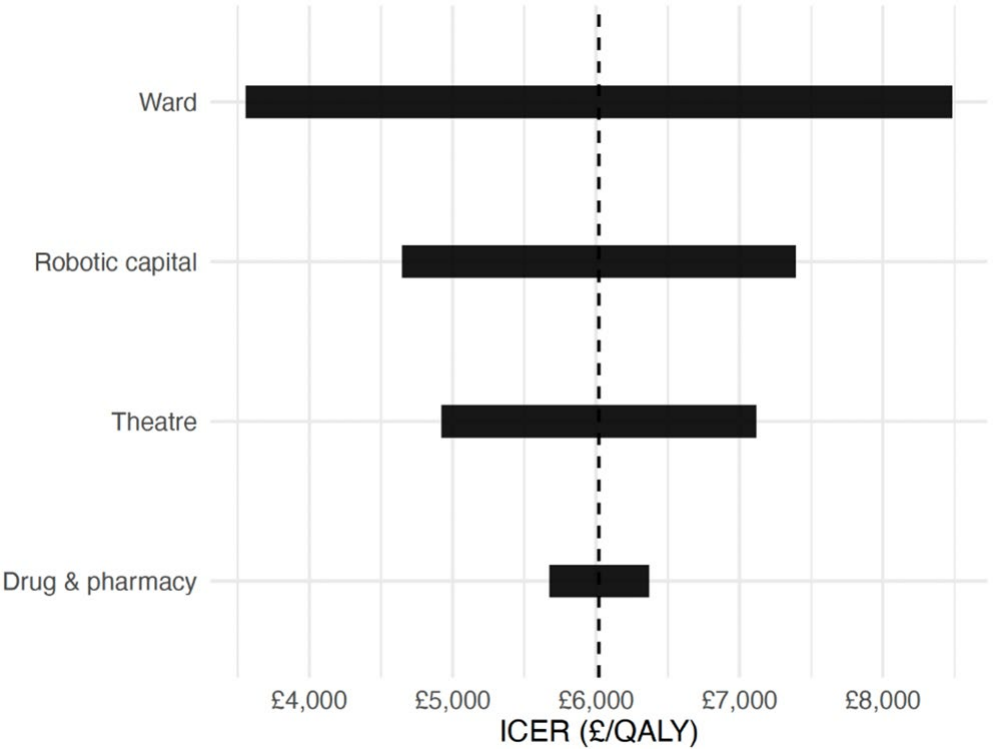
One-way deterministic sensitivity (± 20%). Each bar represents ICER range when that component’s incremental cost varies ± 20%; others held at base

As shown in the Tornado plot (Fig. 1), ward costs exerted the greatest influence on the ICER, reflecting their close association with LOS. Robotic capital costs were the second most influential parameter, followed by theatre costs. Importantly, no plausible variation in individual cost components resulted in an ICER exceeding accepted UK cost-effectiveness thresholds, supporting the robustness of the base-case conclusion.

### Probabilistic sensitivity analysis

**Fig. 2:**
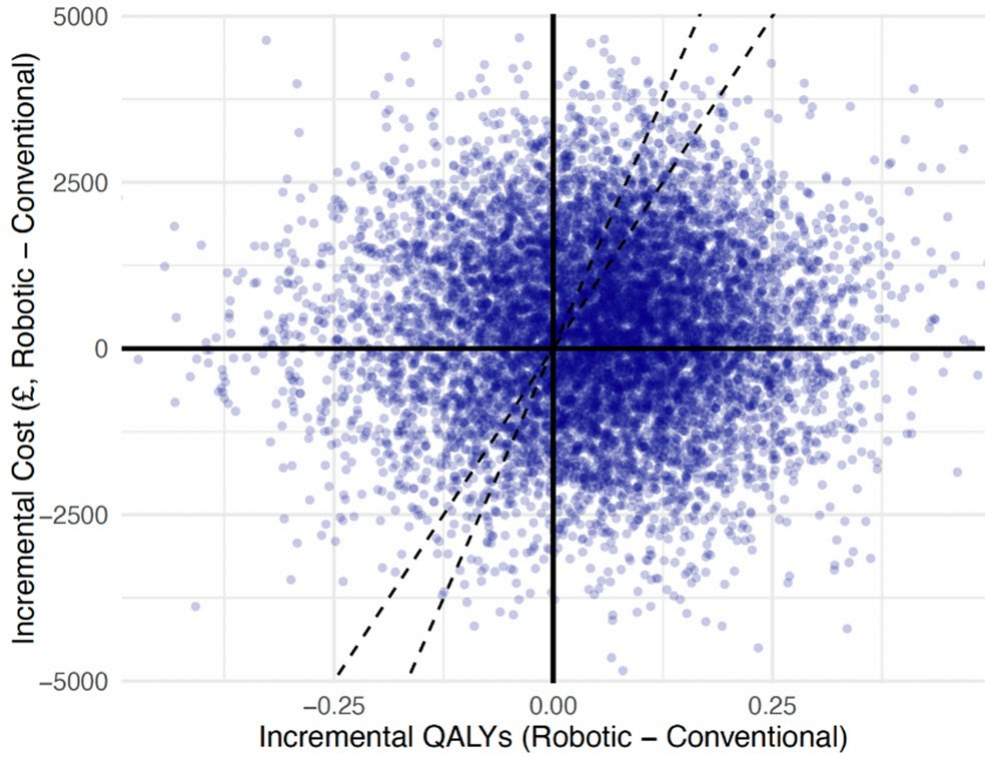
*Cost-effectiveness plane illustrating 10*,*000 bootstrapped cost-effect pairs for RO TKA vs. CO TKA.* Dashed lines = Willingness-to-pay at £20,000 and £30,000 thresholds

PSA was conducted using 10,000 Monte Carlo simulations to jointly explore uncertainty in incremental costs and QALYs. Costs were modelled using gamma distributions with a coefficient of variation of 30%, while QALY gains for each group were modelled using beta distributions derived from observed mean values and 95% confidence intervals. Incremental cost-effect pairs from the PSA are presented in the cost-effectiveness plane (Fig. 2).

The majority of PSA iterations were located in the north-east quadrant, indicating the RO TKA was more effective, but more costly than CO TKA in most simulations. A smaller proportion of simulations crossed into the inferior quadrants, reflecting residual uncertainty around both costs and health outcomes. Importantly, a substantial proportion of simulations fell below the WTP thresholds of £20,000 and £30,000 per QALY, supporting the economic attractiveness of RO TKA across a wide range of plausible parameter values.

**Fig. 3:**
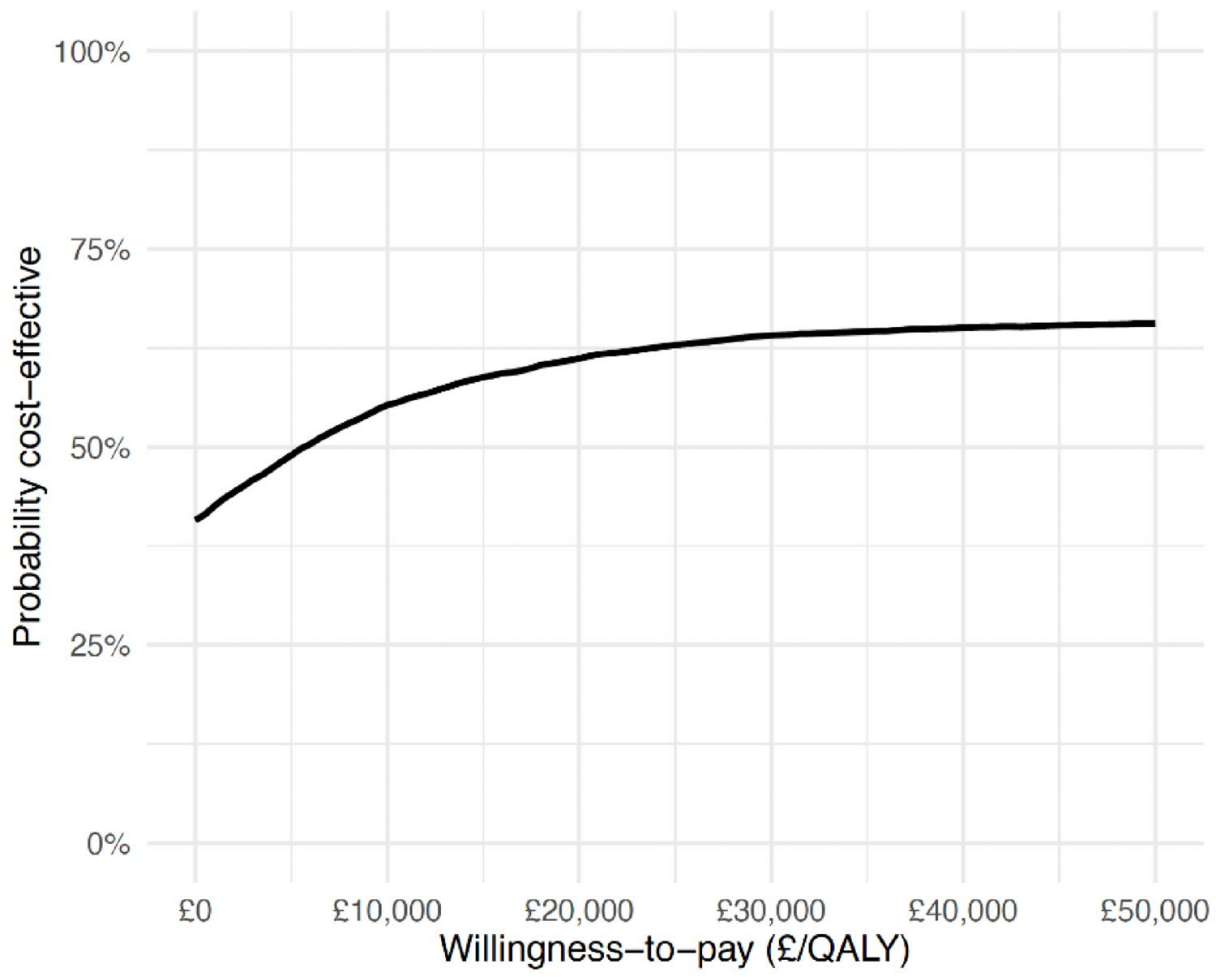
Cost-effectiveness acceptability curve for RO TKA vs. CO TKA. RO TKA = robotic arm-assisted total knee arthroplasty; CO TKA = conventional total knee arthroplasty

The cost-effectiveness acceptability curve (CEAC) (Fig. 3) demonstrates that the probability of RO TKA being cost-effective is approximately 61% at a WTP threshold of £20,000, increasing to approximately 65% at a £30,000 threshold.

## Discussion

This study is the first to undertake detailed micro-costing cost-utility analysis comparing RO TKA with CO TKA in a publicly funded healthcare institution. Leveraging granular in-hospital cost data alongside one-year health-related QoL outcomes, the analysis shows that RO TKA is associated with superior postoperative utility and a favourable incremental cost-effectiveness profile. Although overall in-hospital costs were slightly higher when incorporating robotic capital costs, RO TKA yielded an ICER of £6,019 per QALY gained, substantially below the accepted NICE WTP threshold of £20,000 to £30,000 per QALY. Together, these results indicate that RO TKA is likely to represent a cost-effective option for publicly funded healthcare institutions under the costing assumptions applied in this analysis.

The principal factor driving the economic appeal of RO TKA was lower postoperative resource use, most notably a statistically significant reduction in postoperative LOS in the robotic group. Patients treated with RO TKA had a median LOS of 2.3 days, compared with 3.1 days for those who underwent CO TKA, resulting in direct savings in ward-related costs as well as reduced expenditure on medications and pharmacy services. As inpatient ward costs represent a major proportion of arthroplasty expenditure within the NHS, even modest reductions in LOS can yield a substantial effect on overall cost-effectiveness. This finding is supported by the sensitivity analyses, which consistently identified ward costs as the most influential parameter affecting the ICER across all deterministic scenarios. Our findings are consistent with previous studies showing earlier mobilization and reduced LOS with RO TKA [15, 22, 23]. Hoeffel et al. conducted a systematic review and meta-analysis of studies from 2016 to 2022 comparing RO TKA with CO TKA, and found a statistically significant 14% reduction in LOS by associated with RO TKA (*p* = 0.022), along with higher rates of discharge home and fewer 90-day readmissions, without incurring increasing in-hospital costs [14]. Additionally, a national study of 8,492 primary TKAs performed across all public hospitals in Hong Kong found that RO TKA was associated with a significantly shorter mean LOS compared to navigation-guided and CO TKA (*p* < 0.001), without differences in reoperation or mortality rates, despite the longer operative times [24]. Moreover, comparative analyses have demonstrated that RO TKA is associated with a shorter LOS when compared with computer navigation-guided TKA[25, 26]. These findings suggest that the observed reduction in postoperative resource utilization is not solely attributable to digital alignment assistance.

In contrast, mean theatre-related costs were significantly higher in the robotic group compared to the conventional group (£1,942 vs. £1,657, respectively; *p* < 0.001). This reflects the longer operative times, including robotic setup time. This is consistent with early adoption of robotic technology and aligns with much of the reported data [14, 27, 28]. Moreover, evidence has shown that as robotic workflows mature, a learning curve is observed, with progressive improvements in operative efficiency such that the initial excess in theatre time diminishes, thereby mitigating the long-term economic impact of robotic adoption on theatre costs [29–31].

The CEAC suggests that RO TKA has an estimated 61% probability of being cost-effective at a WTP threshold of £20,000, increasing modestly to 65% at a threshold of £30,000.These probabilities reflect a level of residual uncertainty in the analysis and should be interpreted with appropriate caution. A substantial proportion of this uncertainty is attributable to ward cost savings, which may not fully translate into realisable reductions in fixed departmental costs in all NHS settings. The economic attractiveness of RO TKA will therefore depend on the extent to which institutions are able to convert reductions in LOS into tangible savings through active bed management or care pathway redesign. Nonetheless, the consistently low ICER observed across both deterministic and probabilistic analyses, together with the clustering of simulations within cost-effective regions of the cost-effectiveness plane, supports a cautious interpretation that RO TKA is likely to represent a cost-effective use of healthcare resources within the UK system under the assumptions applied in this study.

From a health economic perspective, RO TKA was associated with a statistically significant improvement in postoperative EQ-5D-3 L utility at one year and a corresponding incremental QALY gain of 0.052 (*p* < 0.001). While this absolute QALY gain may appear modest, it should be interpreted within the context of knee arthroplasty, which is already a very effective intervention associated with substantial baseline improvements in quality of life. Consequently, additional gains attributable to technological innovation are inherently limited, yet remain clinically relevant when considered against established cost-effectiveness thresholds. The between-group difference in postoperative EQ-5D-3 L utility of 0.07 (Cohen’s d = 0.46) falls marginally below the point estimate for minimal clinically important difference (MCID) of 0.085 reported for this instrument in primary knee arthroplasty populations (95% CI 0.042 to 0.127) [21]. However, it sits within the confidence interval for that estimate, and the medium effect size suggests the observed difference is of clinical relevance, albeit one that warrants consideration in the context of longer-term follow-up data. Several mechanisms may plausibly explain the observed improvements in health quality following RO TKA. Enhanced accuracy of component positioning, improved soft tissue balancing and more consistent achievement of target alignment may contribute to reduced pain, improved function and earlier recovery [11, 32, 33]. These technical advantages are particularly relevant in knee arthroplasty, where small deviations in alignment and balance can have a substantial impact on PROMs. All of the RO TKA procedures in the study participants were performed using functional alignment philosophy, which has been purported to improved knee kinematics and functional outcomes [34–38], potentially translating into higher postoperative QoL scores as captured by the EQ-5D-3 L. It is also possible that patient expectations and perceptions surrounding robotic arthroplasty contribute to improved self-reported outcomes, an effect which has been described and warrants further investigation[39].

Existing economic evaluations of RO TKA have predominantly been conducted in private or mixed healthcare systems, often using episodes-of-care costs or modelled assumptions rather than detailed patient-level micro-costing data [40–43]. Studies have reported higher upfront costs associated with robotic technology, but potential downstream savings related to reduced complications and post-care utilization [44–46]. Mont et al. reported, in a propensity-matched analysis of Medicare beneficiaries, that RO TKA was associated with significantly lower 30-, 60- and 90-day episode-of-care costs compared to manual TKA, driven by lower emergency department visits postoperatively, fewer readmissions and fewer home-health visits [47]. In a retrospective analysis, Kolessar et al. found that the introduction of robotic technology led to an increase in the number of PKA procedures being performed at their institution by 190% [48]. Similarly, at our institution, a substantial proportion of knee arthroplasty procedures are performed as PKA, a shift that has also been facilitated by robotic assistance; given the reduced soft-tissue disruption, shorter LOS, reduced morbidity and faster functional recovery associated with PKA [49, 50]. This change in case mix is likely to improve the overall cost-effectiveness of robotic knee arthroplasty at institutions that utilize robotic technology for knee arthroplasty. Our study adds to the existing literature by presenting real-world patient-level cost and outcome data from a publicly funded healthcare institution. This offers pragmatic, policy-relevant evidence for healthcare commissioners and decision-makers evaluating the role and value of robotic TKA within resource-constrained public health systems.

A strength of this study is the comprehensive micro-costing methodology that has been employed, which has allowed the capturing of granular resource utilization across theatre, anaesthesia, ward, pharmacy, diagnostics and robotic-specific cost domains. This approach provides a more accurate reflection of true NHS expenditure than tariff-based or modelled analyses and enhances the external validity of the findings for other high-volume public sector institutions. Additionally, the use of prospectively collected health utility data and established QALY estimation methods strengthen the robustness of the economic evaluation.

There are some limitations that need to be acknowledged. Firstly, this analysis was conducted in a high-volume arthroplasty centre with extensive surgical experience in robotic-assisted surgery. Consequently, the per-case allocation of robotic capital costs may be lower than what is achievable in a lower-volume arthroplasty centre, potentially limiting the generalisability of our results. Also, the one-year time horizon for health utility data collection may underestimate the full extent of the economic and clinical benefits of RO TKA, particularly if improved alignment and soft tissue balance translate into reduced revision rates and sustained functional advantages over time. Longer-term follow up and modelled lifetime analyses will be necessary to capture these potential longer-term benefits. Community-based costs, including outpatient physiotherapy and outpatient follow-up costs were not captured in either cohort, which underestimates total downstream resource utilization in both groups. Furthermore, it should be acknowledged that reductions in LOS do not automatically translate into proportional reductions in direct ward costs within an NHS setting, where nursing staffing and overhead expenditure are predominantly fixed. The ward cost differences reported in this study therefore reflect reductions in per-patient resource utilisation rather than confirmed savings in fixed hospital costs, and appropriate caution should be exercised when interpreting the magnitude of the ward cost differential and its contribution to the ICER.

In conclusion, this study demonstrates that RO TKA delivers superior early health utility outcomes and is likely to achieve cost-effectiveness within a publicly funded healthcare system, despite higher upfront costs associated with robotic assistance. The favourable ICER, robust sensitivity analyses and consistent efficiency gains support the role of RO TKA as a likely clinically and economically viable innovation in knee arthroplasty, under the costing assumptions applied in this NHS setting. Wider applicability will depend on institutional volume, procurement model and the extent to which reductions in LOS translate into direct cost savings at the departmental level. As healthcare systems increasingly prioritise value-based care, high-quality economic evaluations are essential in guiding the rational adoption of emerging surgical technologies. Further long-term studies are required to assess whether the observed benefits are sustained and amplified over the lifetime of the implant.

## Data Availability

The data that support the findings of this study are not publicly available due to confidentiality and/or privacy restrictions. These data were obtained under agreement with organization, which does not permit public sharing. Data may be available from the corresponding author upon reasonable request and with permission of our organization.
